# Boundaries for martensitic transition of ^7^Li under pressure

**DOI:** 10.1038/ncomms9030

**Published:** 2015-08-14

**Authors:** Anne Marie Schaeffer, Weizhao Cai, Ella Olejnik, Jamie J. Molaison, Stanislav Sinogeikin, Antonio M. dos Santos, Shanti Deemyad

**Affiliations:** 1 Department of Physics and Astronomy, University of Utah, 115S 1400E, Salt Lake City, Utah 84112, USA; 2 Quantum Condensed Matter Division, Neutron Sciences Directorate, Oak Ridge National Laboratory, Oak Ridge, Tennessee 37831, USA; 3 HPCAT, Geophysical Laboratory, Carnegie Institution of Washington, Argonne, Illinois 60439, USA

## Abstract

Physical properties of lithium under extreme pressures continuously reveal unexpected features. These include a sequence of structural transitions to lower symmetry phases, metal-insulator-metal transition, superconductivity with one of the highest elemental transition temperatures, and a maximum followed by a minimum in its melting line. The instability of the bcc structure of lithium is well established by the presence of a temperature-driven martensitic phase transition. The boundaries of this phase, however, have not been previously explored above 3 GPa. All higher pressure phase boundaries are either extrapolations or inferred based on indirect evidence. Here we explore the pressure dependence of the martensitic transition of lithium up to 7 GPa using a combination of neutron and X-ray scattering. We find a rather unexpected deviation from the extrapolated boundaries of the hR3 phase of lithium. Furthermore, there is evidence that, above ∼3 GPa, once in fcc phase, lithium does not undergo a martensitic transition.

## Abstract (web summary)

Lithium metal under extreme pressures shows a sequence of structural phase transitions. Here, the authors use neutron scattering and X-ray diffraction techniques under high pressure to expand the experimental phase diagram of lithium, showing an unexpected deviation from existing boundaries.

## Graphical abstract

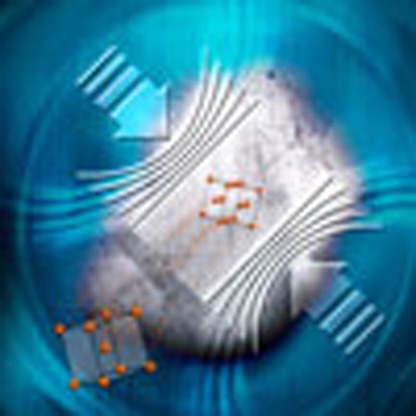

Lithium is the simplest metal that undergoes temperature-driven martensitic transition^1^,^2^,^3^,^4^. These are an important class of diffusion-less structural phase transitions that are integral to controlling the properties of steels and the design of many technologically important materials such as shape memory alloys. Because of its simple electronic structure, studies on the martensitic transition of lithium are of fundamental interest. Under ambient conditions, lithium is a nearly free electron system and has a bcc structure; however, the structures and electronic properties of lithium under compression deviate rapidly from a simple metal^5^,^6^,^7^,^8^,^9^,^10^,^11^,^12^. Like all other alkali metals, lithium undergoes a series of pressure-induced structural phase transitions to low symmetry phases, following an initial pressure-induced transition from bcc to fcc phase at room temperature.

Whilst structures of lithium under pressure have been extensively studied, the mapped boundaries of the martensitic phase transition are limited to pressures below 3 GPa. In this region, lithium adopts the bcc phase at room temperature with a martensitic transition temperature (*M*_s_), *M*_s_|^*P*=0^≈70–75 K, that increases with pressure at a rate of 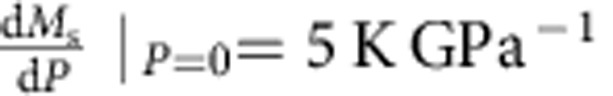
(ref. 3). The low-temperature structure of lithium was initially identified to be hexagonal-close-packed^13^ but later was shown to be samarium-type R-3 m H (166) structure (hR3) with 3 atoms per unit cell^1^,^14^,^15^,^16^,^17^,^18^,^19^.

Lithium is a weak scatterer of both X-rays and neutrons, rendering scattering experiments only feasible in large facilities. Still neutron scattering is, in principle, more appropriate for structural studies of lithium, especially those at high pressure, as it combines a superior penetrating power with an improved scattering performance, when compared with X-rays, given the low atomic number of lithium. Yet, despite recent advances of neutron scattering in diamond anvil cells (DACs), these experiments remain challenging. Natural lithium is composed of 7% ^6^Li, which has a neutron absorption cross section of 940 barn, thus requiring, for neutron studies, isotopically pure ^7^Li (0.0454 barn), which is not commercially available. In addition, its softness and tendency to texture makes the production of both high quality fine powder or large single crystals of lithium extremely difficult, consequently limiting the quality of the structural data obtained, particularly inside a DAC as it requires very small samples. These limitations have led to an under-constrained low-temperature/high-pressure structural phase of lithium built mostly by extrapolation from higher temperature structural data or based on indirect evidence such as pressure dependence of its superconducting transitions^6^,^11^. Earlier experiments only reported the superconductivity of lithium above ∼20 GPa with an initial positive and steep (d*T*_c_)/(d*P*). Later the superconducting phase diagram of lithium was revised with the discovery of ambient pressure superconductivity with *T*_c_ ∼0.4 mK^20^ and lower boundaries of the superconducting phase transition under pressure shifted down to ∼16 GPa leaving an unexplored region of phase diagram for superconducting properties between ambient-16 GPa (ref. 12). On the basis of these results, it was suggested that the emergence of the pressure-induced superconductivity of lithium is correlated with its structural boundaries at low temperature^11^, with the fcc phase favouring superconductivity and the hR3 phase suppressing it. This, together with the higher temperature structural data leads to the conclusion that, akin to the bcc phase, also fcc lithium will undergo a martensitic phase transformation to the hR3 phase, extending the possible stability field of the hR3 phase to ∼20 GPa (or at least 16 GPa). It is thus clear that understanding the superconducting behaviour and other low-temperature properties of lithium as a function of pressure, relies first and foremost on an accurate picture of its low-temperature phase boundaries^1^,^2^,^4^,^21^. In this study, we used state of art neutron and X-ray diffraction techniques under high pressure to expand the experimental phase diagram of lithium.

## Results

### Structural analysis of martensitic transition

**Figure 1:**
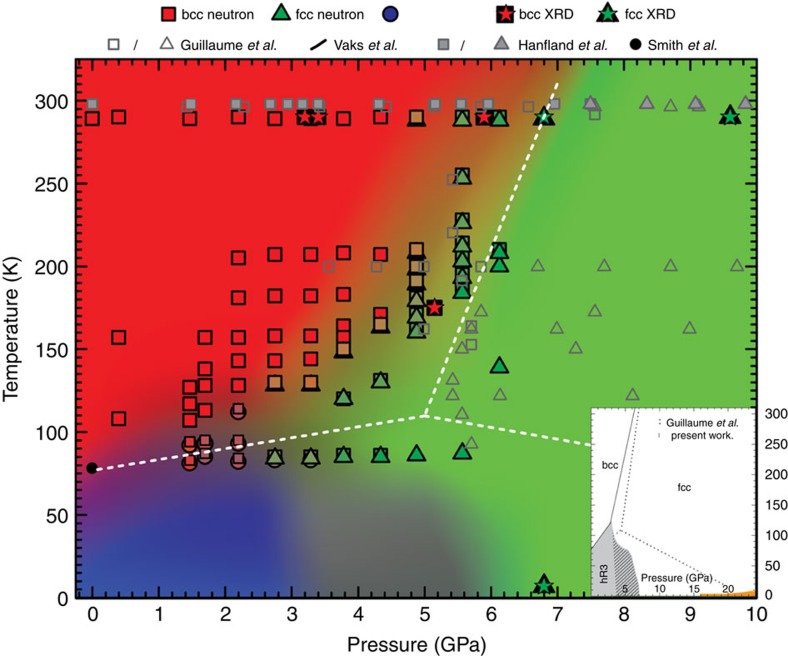
PT phase diagram of lithium from neutron and X-ray diffraction. Phase boundaries obtained from data collected while cooling. Background shades are used to highlight the regions of the PT phase diagram of lithium in which different structures are observed. Symbols designate different structures. Symbol overlap indicates the presence of mixed phases. The blue region here indicates two possibilities for the fcc/hR3 phase boundary, one boundary in which a transition from fcc to hR3 is not supported and one boundary drawing the upper limit. The inset shows the proposed structural boundaries of martensitic phase transition in lithium overlaid with previous reports. Orange region represents the superconducting region^9^,^11^,^12^,^31^.

**Figure 2:**
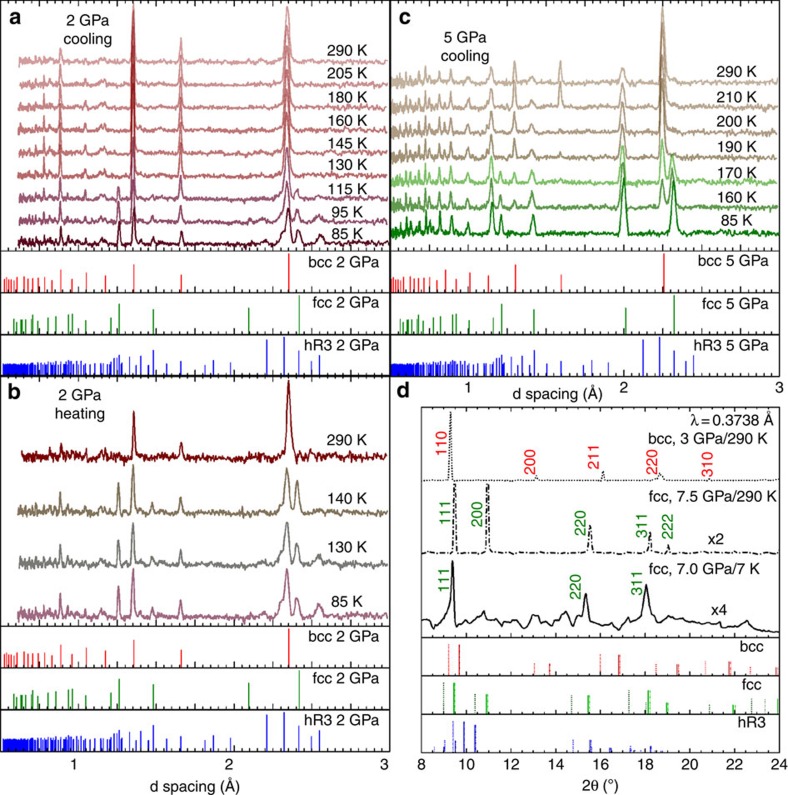
Neutron and X-ray diffraction pattern at selected pressures. (**a**) and (**b**) Neutron diffraction pattern of lithium at 2 GPa for cooling and heating, respectively. The calculated peaks for the structures for this pressure are plotted below the spectra. The conversion on cooling to hR3 is evident, and hysteresis is observed. The refined *a* lattice parameter for the bcc phase at room temperature is 3.323±0.002 Å and at base temperature is 3.322±0.002 Å. For the hR3 phase the calculated lattice constants at base temperature are *a*=2.959±0.004 Å and *c*=21.726±0.001 Å. (**c**) The neutron diffraction at 5 GPa shows no indication of hR3 phase down to the lowest temperature. The lattice parameter a for the bcc phase at room temperature is 3.196±0.001 Å, and for the fcc phase at base temperature *a*=4.002±0.003 Å. (**d**) Structures of lithium at 3 and 7 GPa measured by X-ray in a DAC shows the presence of fcc phase to 7 K. Intensities are scaled for each data set. The dotted and solid lines show the location of the peaks at 3 and 7 GPa, respectively.

All structures were mapped by neutron scattering and X-ray angle dispersive diffraction using the SNAP instrument at the Spallation Neutron Source and by the High Pressure Collaborative Access Team (HPCAT) at the Advanced Photon Source (APS), respectively (Methods section). Figure 1 shows the revised phase diagram of lithium based in this study together with a selection of the data points previously reported by various groups. Supplementary Fig. 1 shows the equation of state (EOS) of lithium at room temperature with neutron and X-ray, compared with previously reported curve for natural lithium. In Supplementary Fig. 2 we plot the low-temperature EOS of lithium at 80 K in which bcc and hR3 phases coexist. The presented data represents the first detailed high resolution structural analysis of the martensitic transition of lithium under pressure, including studies of the thermal hysteresis of the structural transformations (Fig. 2 and Supplementary Fig. 3). The observed bcc-hR3 phase boundary below 3 GPa is in agreement with those measured previously by Vaks *et al.*^3^ using acoustic emission method. In a narrow pressure region between 2.5 GPa<*P*<3.5 GPa, the co-existence of a very small amount of hR3 with fcc and bcc phase is possible. Above 3.5 GPa, the bcc phase partially or completely converts to fcc, which remains to the lowest temperatures achieved.

### Mixed phases under pressure

**Figure 3:**
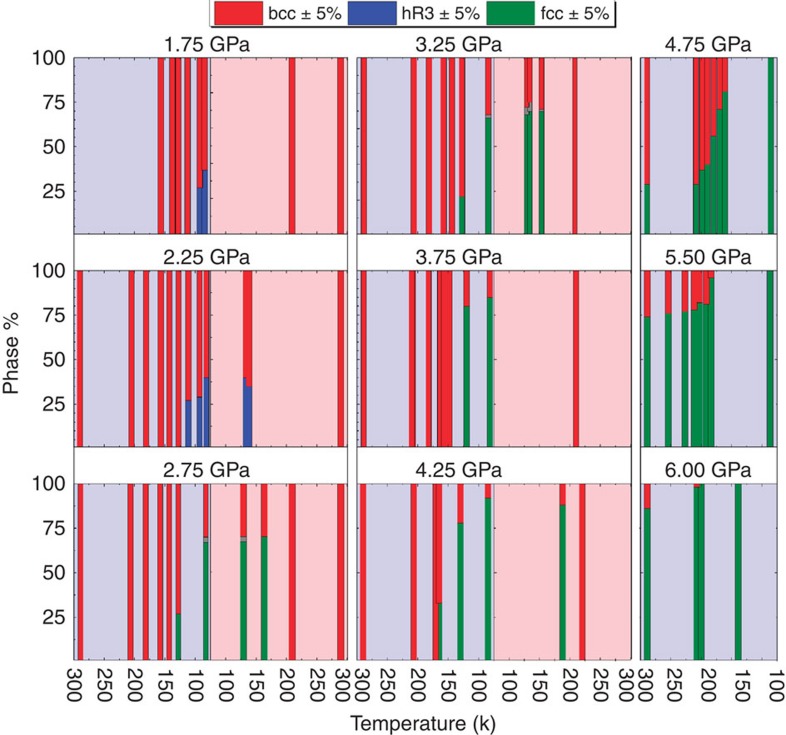
The calculated phase fractions of each structure of lithium. Phase fractions are presented in order during each isobaric cooling and subsequent heating. The grey area reported for 2.75 and 3.25 GPa are representative of areas with peaks of hR3 phase that appear as weak shoulders on characteristic bcc and fcc peaks. This could be indicative of a co-existence of bcc/fcc/hR3 or other phase. The slope of the hR3-fcc boundary in the region between 3.5 and 7 GPa below 80 K cannot be well defined by the current data. It should be noted, however, that at these pressures, once some percentage of the sample undergoes the transition to fcc, that percentage does not decrease on further cooling to 80 K. This raises the possibility that bcc/fcc/hR3 co-exist in these pressure–temperature regions, and it is only the bcc that transforms to hR3, and the fcc percentage is stable or increasing on cooling. It remains an open question if the transformation of fcc to hR3 on cooling is possible.

The phase fractions measured during each isobaric cooling show an increase in the percentage of fcc phase down to the lowest measured temperatures (Fig. 3). On the other hand, the percentage of bcc phase decreases as result of isobaric cooling or during isothermal compression as it transforms into the hR3 and/or fcc structures. This suggests that only the bcc phase will convert to a fcc or hR3 phase and conversion of fcc to hR3 on cooling or vice versa is not explicitly supported by the current experimental observations. Transition from bcc to hR3 is shown theoretically to be energetically favourable based on Hume-Rothery arguments^1^. However, similar arguments are not shown to apply similarly to support the transition of fcc phase to hR3 or another structure. Specifically, this is seen in pressures above 5 GPa, at which the entire sample converts to fcc with moderate cooling; and on further cooling, no indication of hR3 phase is observed down to the lowest achievable temperatures.

### Complementary X-ray diffraction

Since achieving temperatures lower than ∼85 K is currently not possible in high-pressure neutron studies, to further investigate the possibility of conversion from fcc to hR3, a complementary set of data at ∼7 GPa down to ∼7 K were collected using *in-situ* X-ray angle dispersive diffraction at APS. This measurement confirms that ^7^Li is already in the fcc form at room temperature, and that remains the only observed phase down to ∼7 K, with no indication of any phase transition during cooling (Fig. 2d). These observations lead us to conclude that, once in fcc phase, ^7^Li is stable in the pressure ranges studied and unlike what is known for the bcc phase, it will not undergo the temperature-driven martensitic phase transition. This confines the boundaries of the martensitic transition to pressures in which the bcc phase is still present and for a maximum of hR3 phase expected to not exceed the proportion of bcc present at the lowest measured temperature (<15% at 3.75 GPa and <10% at 4.25 GPa). It is notable that this conclusion could not be made from the previously reported data alone, also that there is agreement in the regions in which the proposed phase diagram and previous works overlap.

## Discussion

According to the previous phase diagram of natural lithium, in the fcc phase, 
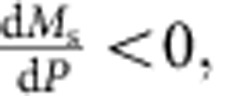
and there is a triple point near ∼110 K and ∼5 GPa (dotted line in Fig. 1 inset). Should this be the case, several of the data points taken at the base temperature (∼85 K) above 3.5 GPa would show the presence of hR3 phase, which is not supported by the current experiments (Figs 2 and 3). On the basis of the updated phase diagram, the triple point is shifted to 115 K and 3 GPa. While isobaric coolings, monitored via *in-situ* neutron scattering, have been used to build the proposed phase diagram, measurements on isobaric heatings were also performed at selected pressures (Fig. 2). The martensitic transition temperature of lithium exhibits hysteresis and on heating, the percentage of hR3 phase does not decrease up to 20 K above *M*_s_ (Fig. 3). Furthermore, some fraction of the hR3 phase is still observed, albeit at lower percentages, up to 30 K above *M*_s_. This is consistent with the findings of Krystian and Pichl^22^,^23^ and Smith *et al.*^16^,^24^. The hR3 phase can be visualized as a sequence of specific stacking of fcc and hcp and the difference in the free energy of fcc and hR3 phases even under ambient pressure is minute (∼1 meV^5^). At pressures in the range between 2.5 GPa<*P*<3.5 GPa, bcc ^7^Li cools into a possible mixture of three phases, dominated by a bcc/fcc mixture with the possibility of hR3 phase also present. Interestingly, on heating, the percentage of bcc decreases while the fcc phase fraction grows, which may indicate the metastability of the hR3 phase in this regime, until the sample reverts back to the ambient temperature phase of 100% bcc. Above 3.5 GPa, ^7^Li cools into a bcc/fcc mixture, and on heating, the bcc phase does recover but only above 20 K of the *M*_s_ on cooling (for example, Fig. 3, 4.25 GPa). This supports the previous reports by Krystian and Pichl^22^,^23^ and Smith *et al.*^16^,^24^ that lithium recovers from the martensitic transition into a mixture of bcc and hR3 phase with possible vestiges of fcc phase.

In summary, revised boundaries for the martensitic transition of ^7^Li are presented. The proposed phase diagram demonstrates new features: a triple point (bcc/fcc/hR3) shifted to a lower pressure of ∼3 GPa; the low-temperature boundary of the martensitic phase transition confined to pressures <5 GPa; and, up to 7 GPa, the fcc phase remains stable down to the lowest measured temperatures without indication of a martensitic phase transition. This opens the possibility for new structural boundaries of lithium below 80 K and at higher pressures, specifically where it was previously thought to be hR3, 7 GPa<*P*<20 GPa.

## Methods

### High-pressure neutron scattering experiments

*In-Situ* diffraction patterns of a polycrystalline sample of 99.9% isotopically pure ^7^Li, were collected using time-of-flight neutron scattering at SNAP. The isotopically pure sample was enriched in ORNL allowing neutron experiments (samples from the same batch were also used for later X-ray diffraction studies in a DAC). The data were collected using a neutron wavelength band centred at 2.15 Å, allowing access to Bragg reflections between 0.7 and 3 Å. Pressure was applied to the sample by means of a Paris–Edinburgh press fitted with cubic boron nitride anvils. Before loading in the press, the sample was sealed in a TiZr null scattering alloy gasket inside a helium glovebox to avoid exposure to atmospheric moisture. The sample was cooled via a custom-made liquid nitrogen cooling device connected directly to the anvils allowing measurements in the 83 K<*T*<320 K range. Complementary X-ray measurements on the same sample were done in HPCAT at 7 GPa from room temperature to 7 K in a DAC. For neutron studies, pressure at each point was determined using the known EOS of bcc lithium at room temperature^5^,^25^,^26^. The pressure was increased at room temperature and temperature-dependent data were taken on isobaric paths. Each pressure/temperature data point were collected in ∼50 min time exposure, after the sample reached thermal equilibrium. While martensitic transitions occur in short time scales, the long exposure time are necessary owing to the weak coherent scattering cross section of lithium. At each pressure the sample was incrementally cooled to the base temperature (∼83 K). Additional data sets were collected on heating to assess the hysteretic nature of the phase transitions. Following each temperature sweep, the sample was annealed to room temperature before a subsequent pressure increase. All the reported phases and transition temperatures used in assembling the proposed phase diagram were extracted from data collected while cooling.

### High-pressure X-ray diffraction experiments

To better define the boundaries of the martensitic transition, we acquired a supplemental set of data points on lithium using high resolution X-ray angle dispersive diffraction in a DAC. Lithium is a very weak X-ray scatterer and thus high-pressure diffraction studies on its structures require high brightness, only accessible at a large facility such as the APS. These experiments were done using a 33 keV X-ray beam at the HPCAT beamline. A sample of lithium was coated with mineral oil, to protect the diamonds, with no other pressure medium. This was loaded in a DAC fitted with a rhenium gasket together with a small chip of NaCl as pressure marker. The initial data were collected at room temperature to confirm the bcc–fcc transition as a function of pressure. A new sample of natural lithium was pressurized to ∼3 GPa at room temperature, then transferred to a continuous flow liquid helium cryostat with 3" diameter Mylar windows and cooled to 7 K. Owing to thermal effects during cooling, the pressure on the sample increased to ∼5.1 and 7.4 GPa at 175 and 7 K, respectively. The DAC was rotated by 20° at a rate of 0.25° s^−1^ and the data were integrated in 83 s exposure time. The pressure was determined using the EOS of NaCl at each point^27^,^28^.

### Structural analysis

The known crystallographic information files of lithium from Inorganic Crystal Structure Database were used to identify the present phases of lithium^29^,^30^ at each pressure/temperature. Dioptas software v. 0.2.3 (2014) was used to analyse the X-ray data.

## Additional information

**How to cite this article:** Schaeffer, A. M. *et al.* Boundaries for martensitic transition of ^7^Li under pressure. *Nat. Commun.* 6:8030 doi: 10.1038/ncomms9030 (2015).

## Supplementary Material

Supplementary Information Supplementary Figures 1-3 and Supplementary Reference
