# Supplementary material for: Boundaries for martensitic transition of 7Li under pressure
Source: Nat Commun. 2015 Aug 14;6:8030. doi: 10.1038/ncomms9030 (PMC4557344; doi:10.1038/ncomms9030)
Supplement: Supplementary Information — Supplementary Figures 1-3 and Supplementary Reference [file ncomms9030-s1.pdf]

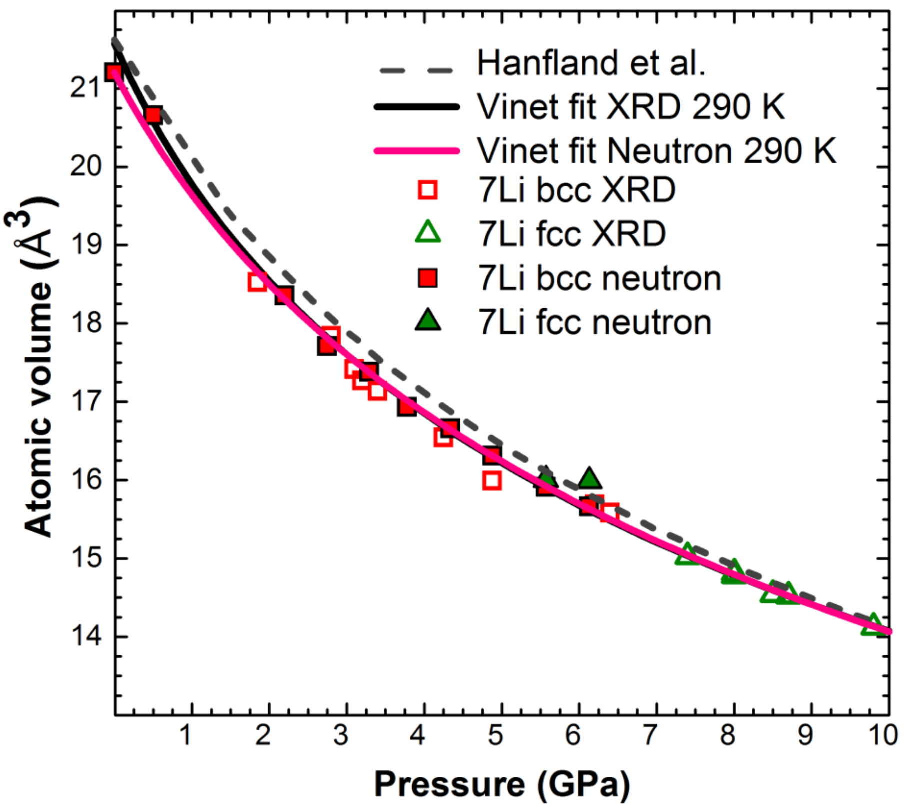

Supplementary figure 1: Equation of state for ^{7}Li measured at room temperature

Squares and triangles represent the experimental data points by neutron scattering and x-ray diffraction in bcc and fcc phase respectively. Open symbols are X-ray data. Dashed line is previously reported EOS for natural lithium[1]. Solid lines are Vinet fits to Neutron and X-ray data.

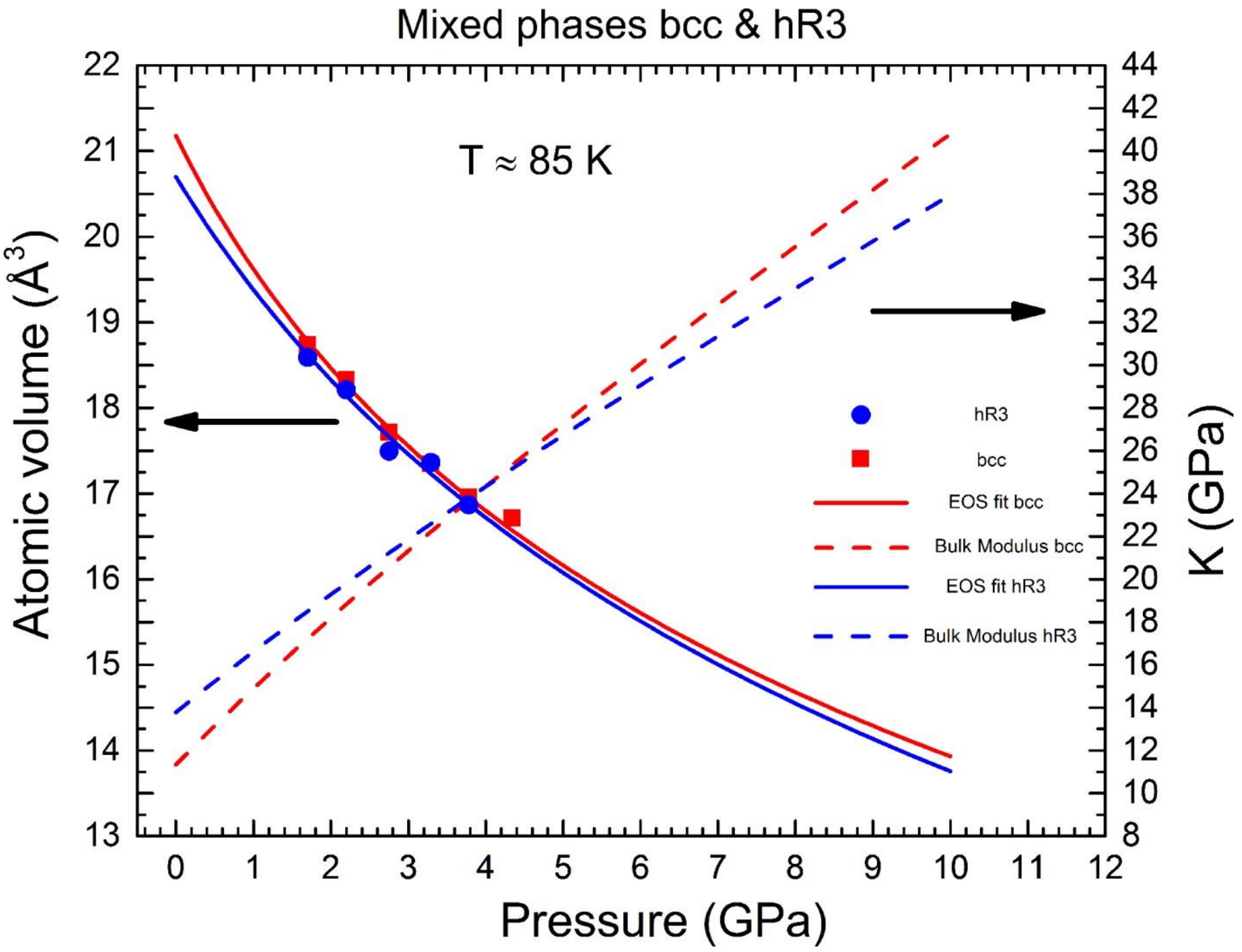

Supplementary figure 2: Equation of state and bulk modulus of lithium at 85 K

Equation of state fits and calculated bulk modulus ($K = (\delta V/\delta P)_T$) for ^{7}Li at base temperature (~80-85 K), in which bcc and hR3 phases coexist, form neutron data. Solid squares and circles are experimental data points of the present study in bcc and hR3 phase respectively.

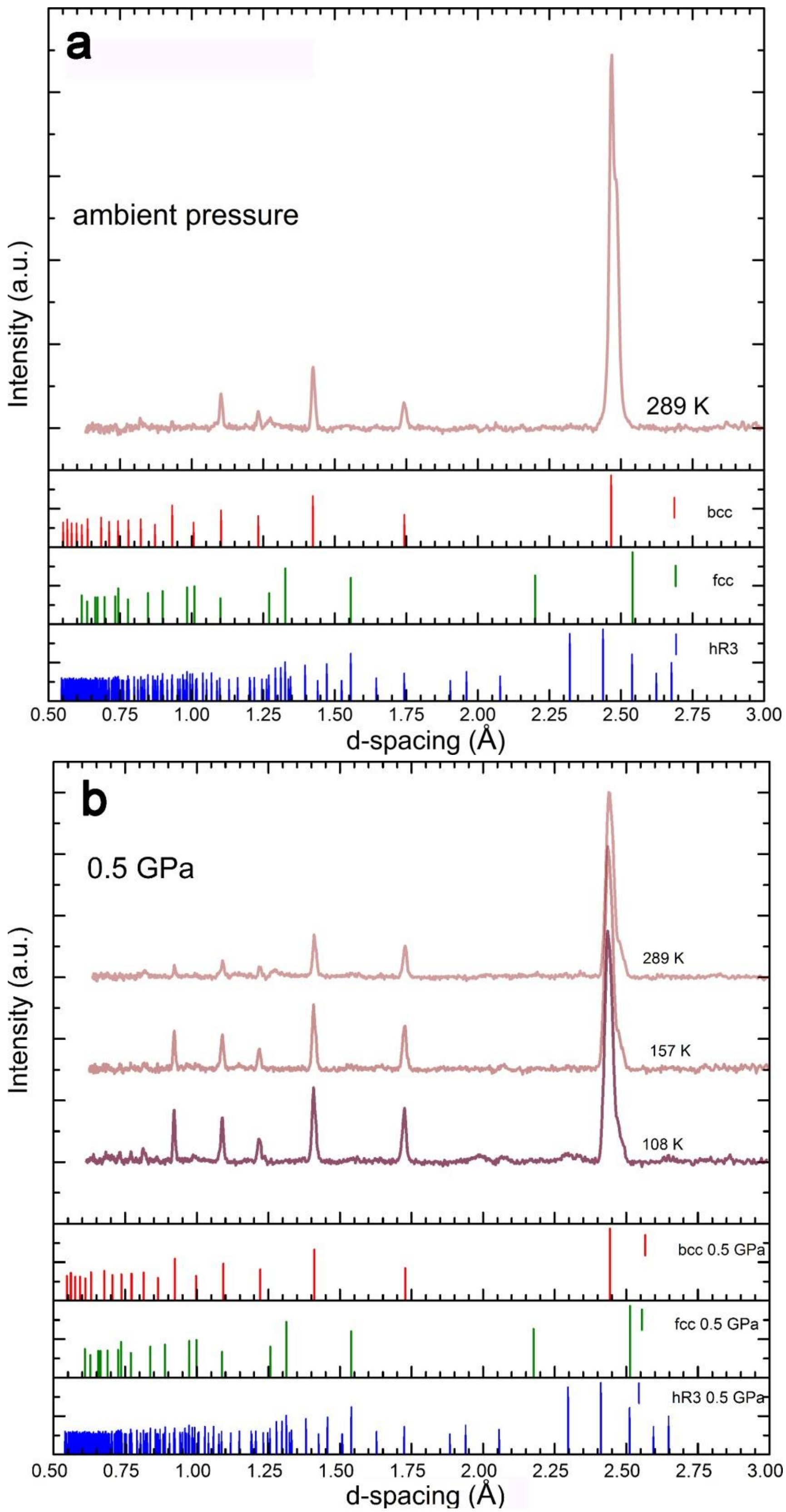
a
ambient pressure
289 K
bcc
fcc
hR3
Intensity (a.u.)
0.50
0.75
1.00
1.25
1.50
1.75
2.00
2.25
2.50
2.75
3.00
d-spacing (Å)
b
0.5 GPa
289 K
157 K
108 K
bcc 0.5 GPa
fcc 0.5 GPa
hR3 0.5 GPa
Intensity (a.u.)
0.50
0.75
1.00
1.25
1.50
1.75
2.00
2.25
2.50
2.75
3.00
d-spacing (Å)

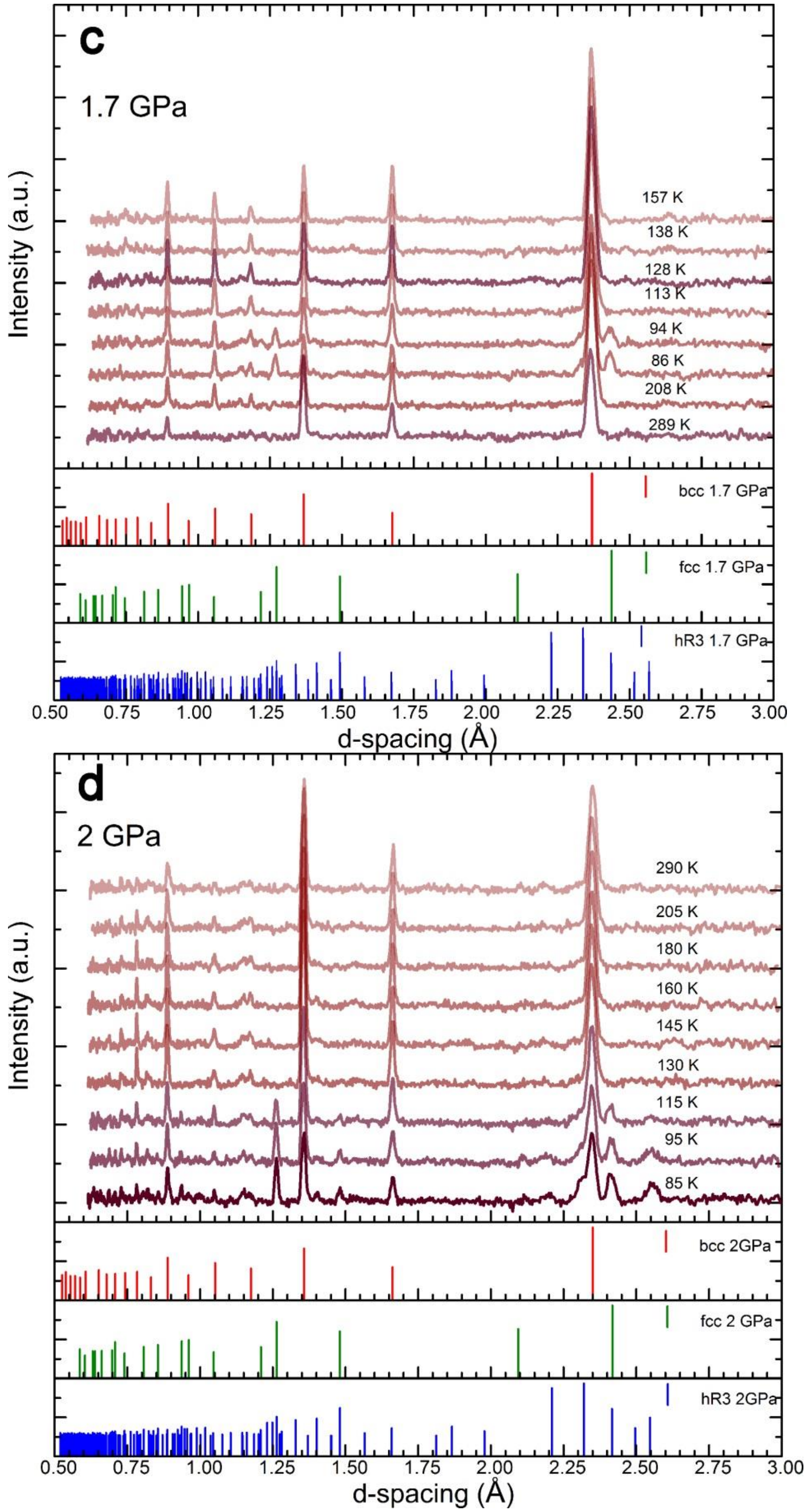
c
1.7 GPa
Intensity (a.u.)
157 K
138 K
128 K
113 K
94 K
86 K
208 K
289 K
bcc 1.7 GPa
fcc 1.7 GPa
hR3 1.7 GPa
0.50 0.75 1.00 1.25 1.50 1.75 2.00 2.25 2.50 2.75 3.00
d-spacing (Å)
d
2 GPa
Intensity (a.u.)
290 K
205 K
180 K
160 K
145 K
130 K
115 K
95 K
85 K
bcc 2GPa
fcc 2 GPa
hR3 2GPa
0.50 0.75 1.00 1.25 1.50 1.75 2.00 2.25 2.50 2.75 3.00
d-spacing (Å)

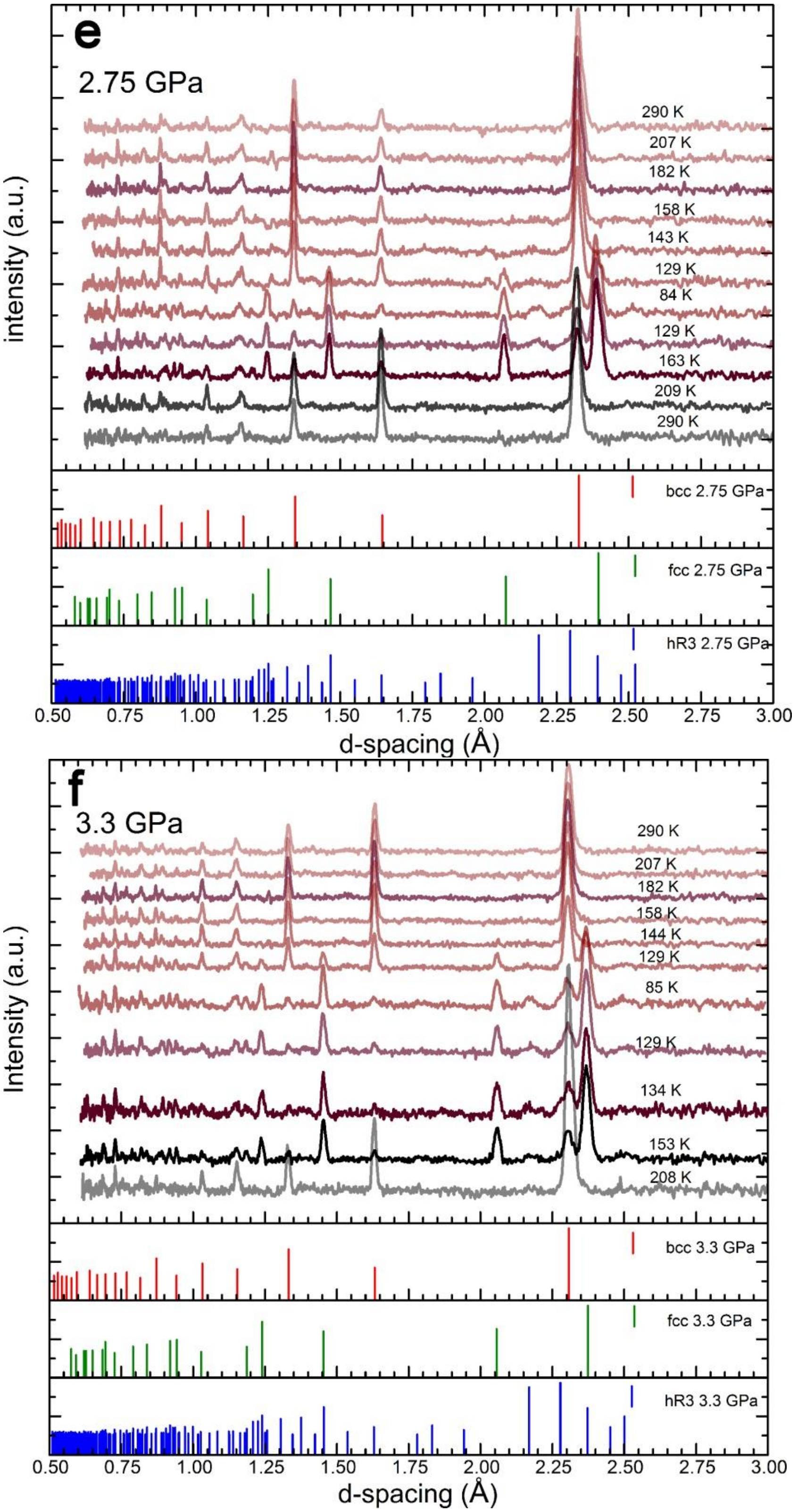
e
2.75 GPa
290 K
207 K
182 K
158 K
143 K
129 K
84 K
129 K
163 K
209 K
290 K
intensity (a.u.)
bcc 2.75 GPa
fcc 2.75 GPa
hR3 2.75 GPa
0.50 0.75 1.00 1.25 1.50 1.75 2.00 2.25 2.50 2.75 3.00
d-spacing (Å)
f
3.3 GPa
290 K
207 K
182 K
158 K
144 K
129 K
85 K
129 K
134 K
153 K
208 K
Intensity (a.u.)
bcc 3.3 GPa
fcc 3.3 GPa
hR3 3.3 GPa
0.50 0.75 1.00 1.25 1.50 1.75 2.00 2.25 2.50 2.75 3.00
d-spacing (Å)

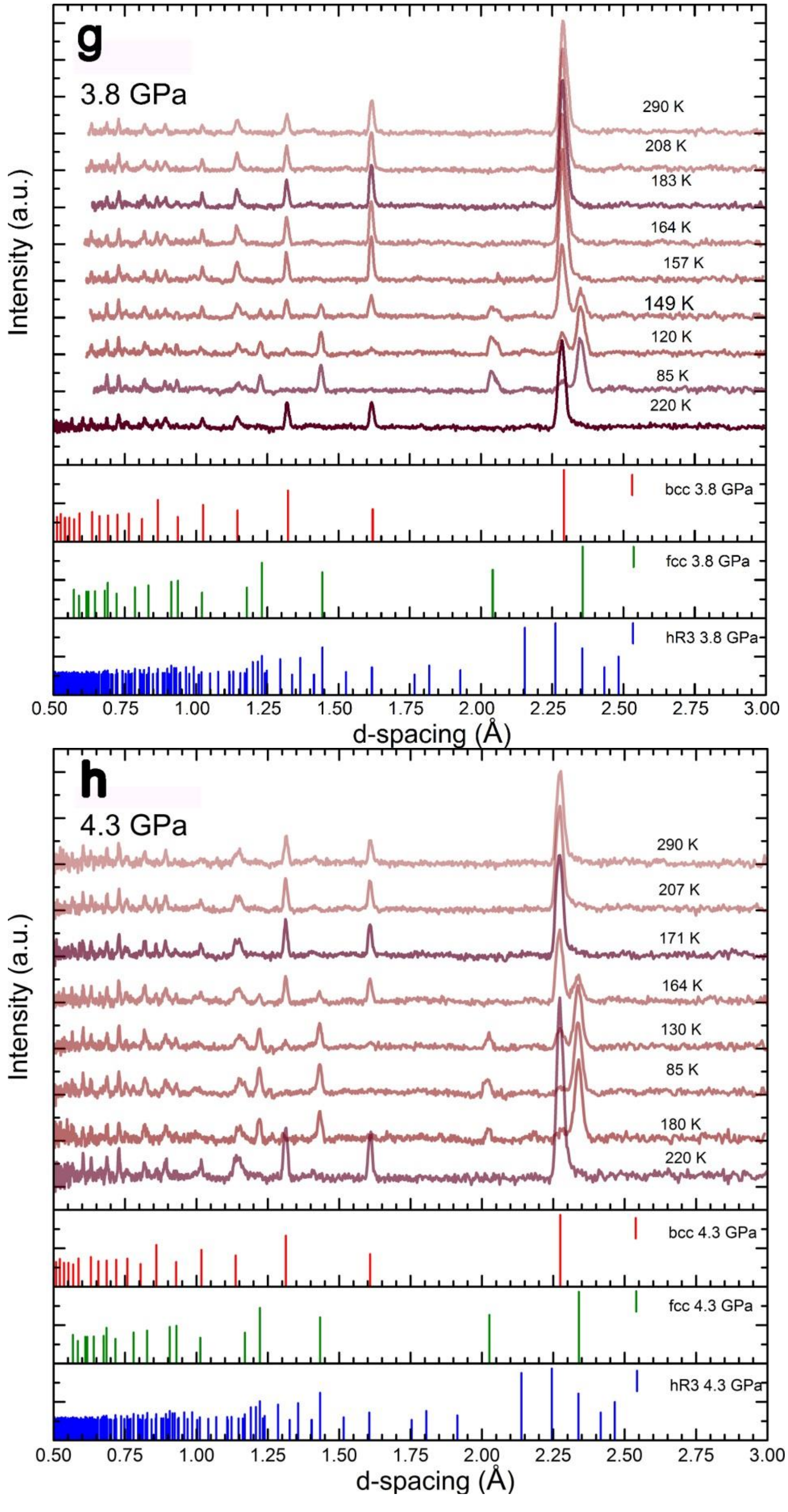

g
3.8 GPa
290 K
208 K
183 K
164 K
157 K
149 K
120 K
85 K
220 K
bcc 3.8 GPa
fcc 3.8 GPa
hR3 3.8 GPa
Intensity (a.u.)
d-spacing (Å)
0.50
0.75
1.00
1.25
1.50
1.75
2.00
2.25
2.50
2.75
3.00
h
4.3 GPa
290 K
207 K
171 K
164 K
130 K
85 K
180 K
220 K
bcc 4.3 GPa
fcc 4.3 GPa
hR3 4.3 GPa
Intensity (a.u.)
d-spacing (Å)

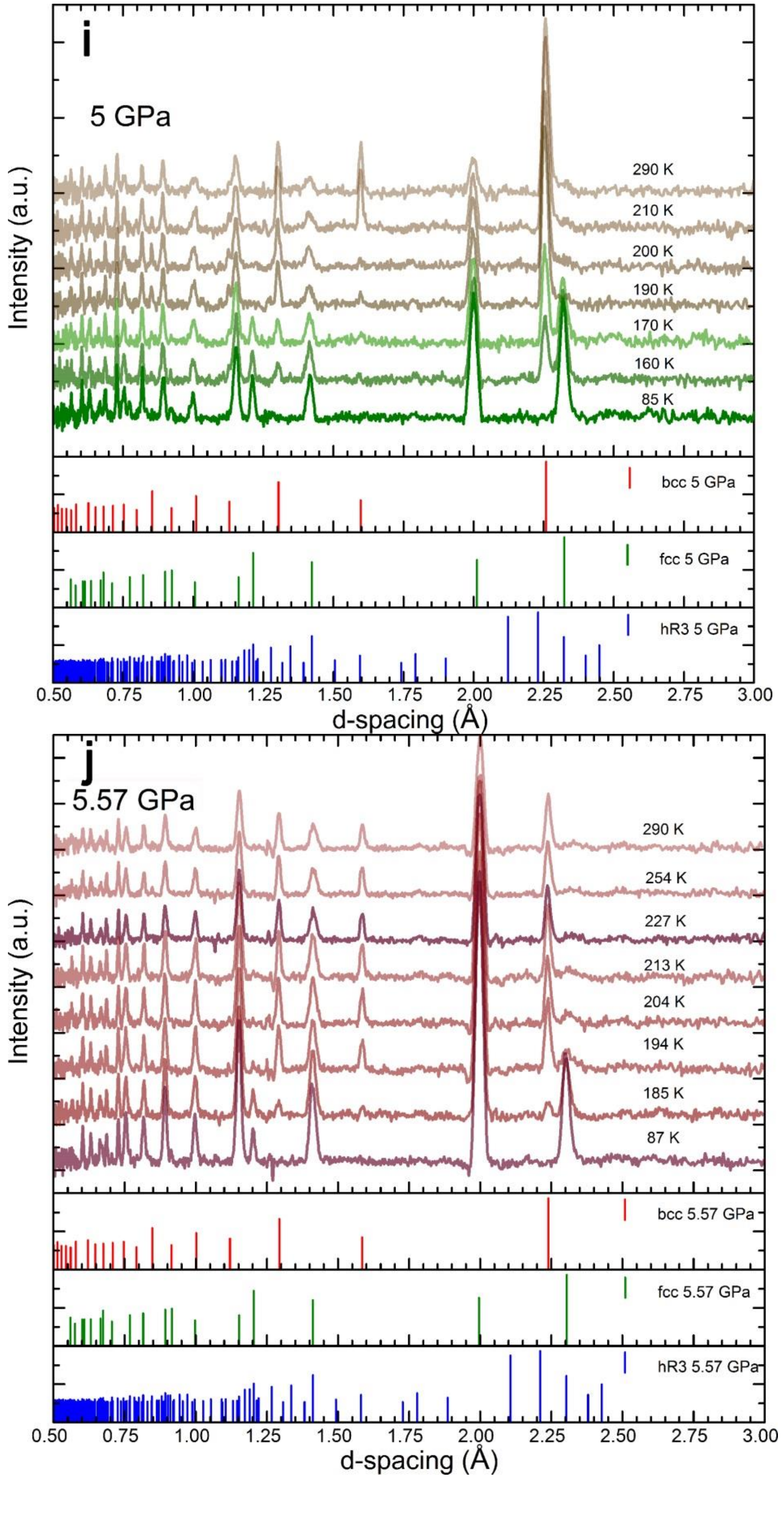
i
5 GPa
290 K
210 K
200 K
190 K
170 K
160 K
85 K
bcc 5 GPa
fcc 5 GPa
hR3 5 GPa
Intensity (a.u.)
d-spacing (Å)
0.50 0.75 1.00 1.25 1.50 1.75 2.00 2.25 2.50 2.75 3.00
j
5.57 GPa
290 K
254 K
227 K
213 K
204 K
194 K
185 K
87 K
bcc 5.57 GPa
fcc 5.57 GPa
hR3 5.57 GPa
Intensity (a.u.)
d-spacing (Å)
0.50 0.75 1.00 1.25 1.50 1.75 2.00 2.25 2.50 2.75 3.00

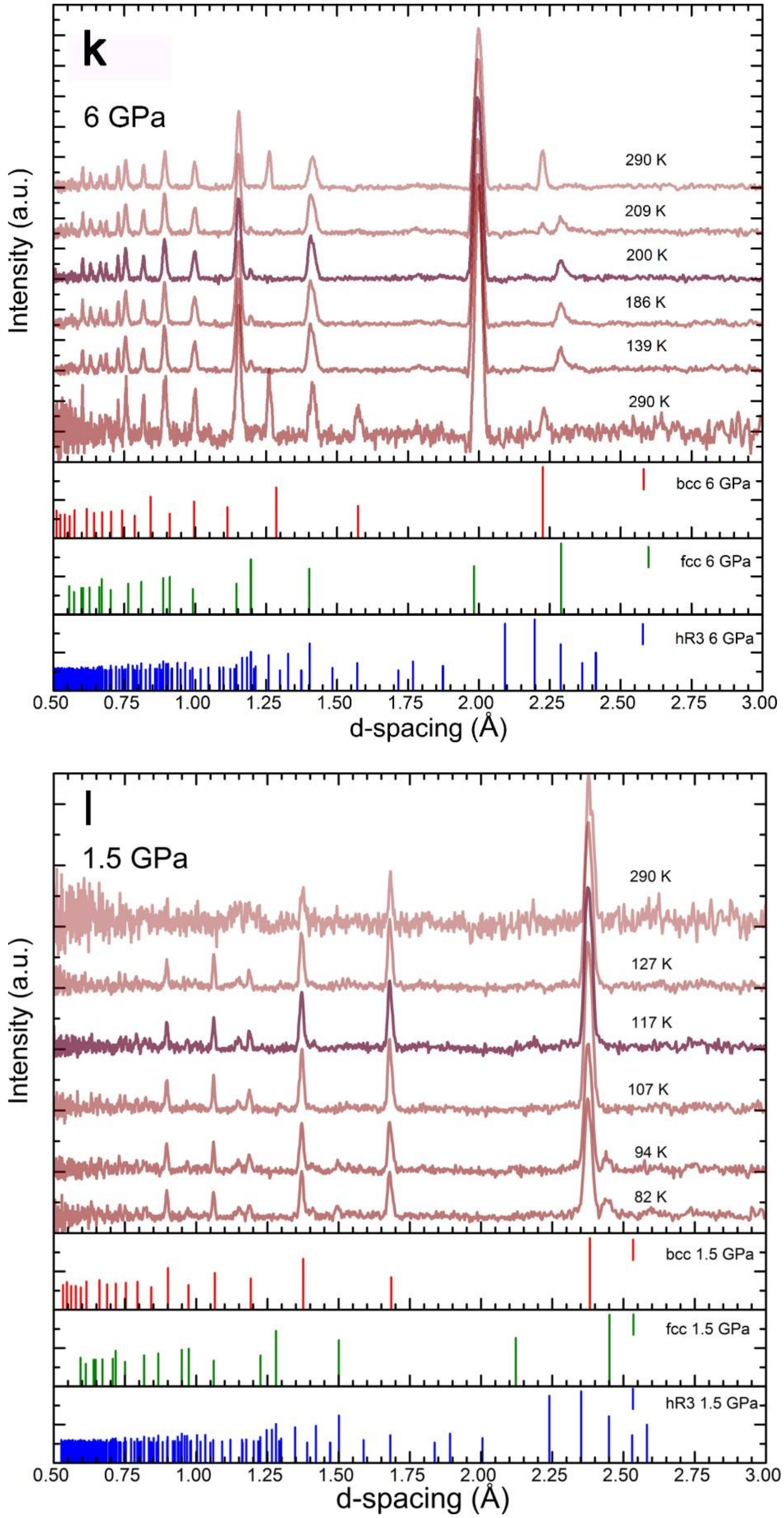

Supplementary figure 3: Neutron diffraction patterns for all pressures and temperatures

a-k) data taken during compression l) data taken during decompression.

Supplementary Reference

1 Hanfland, M., Loa, I., Syassen, K., Schwarz, U. & Takemura, K. Equation of state of lithium to 21 GPa. *Solid State Communications* **112**, 123-127, doi:http://dx.doi.org/10.1016/S0038-1098(99)00322-1 (1999).
